# The Wheat NAC Transcription Factor TaNAC2L Is Regulated at the Transcriptional and Post-Translational Levels and Promotes Heat Stress Tolerance in Transgenic Arabidopsis

**DOI:** 10.1371/journal.pone.0135667

**Published:** 2015-08-25

**Authors:** Weiwei Guo, Jinxia Zhang, Ning Zhang, Mingming Xin, Huiru Peng, Zhaorong Hu, Zhongfu Ni, Jinkun Du

**Affiliations:** 1 State Key Laboratory for Agrobiotechnology, Key Laboratory of Crop Heterosis and Utilization (MOE), Beijing Key Laboratory of Crop Genetic Improvement, China Agricultural University, Beijing, 100193, China; 2 National Plant Gene Research Centre (Beijing), Beijing, 100193, China; University of Heidelberg, GERMANY

## Abstract

Heat stress poses a serious threat to global crop production. In efforts that aim to mitigate the adverse effects of heat stress on crops, a variety of genetic tools are being used to develop plants with improved thermotolerance. The characterization of important regulators of heat stress tolerance provides essential information for this aim. In this study, we examine the wheat (*Triticum aestivum*) NAC transcription factor gene *TaNAC2L*. High temperature induced *TaNAC2L* expression in wheat and overexpression of *TaNAC2L* in *Arabidopsis thaliana* enhanced acquired heat tolerance without causing obvious alterations in phenotype compared with wild type under normal conditions. *TaNAC2L* overexpression also activated the expression of heat-related genes in the transgenic Arabidopsis plants, suggesting that TaNAC2L may improve heat tolerance by regulating the expression of stress-responsive genes. Notably, TaNAC2L is also regulated at the post-translational level and might be degraded via a proteasome-mediated pathway. Thus, this wheat transcription factor may have potential uses in enhancing thermotolerance in crops.

## Introduction

Hexaploid wheat (*Triticum aestivum* L. AABBDD), a major food crop, provides dietary carbohydrates for more than one-third of the world’s population [1, 2]. In plants, high temperature can induce heat stress, which triggers a wide range of responses, altering gene expression, cellular metabolism, growth, development, yield, and quality. Profiling of the response of the wheat transcriptome to heat [3, 4] identified heat-stress-inducible genes encoding heat shock proteins (HSPs), heat shock factors (HSFs), and other transcription factors. The analysis also found heat-responsive genes that participate in phytohormone biosynthesis/signaling, calcium and sugar signaling, metabolism of RNA and ribosomes, and primary or secondary metabolism.

Transcription factors function as important players in stress responses by binding to *cis*-elements in the promoter regions of target genes. The plant-specific NAC transcription factors have a characteristic, conserved N-terminal region known as the NAC domain and include petunia NAM (for no apical meristem) and Arabidopsis ATAF1, ATAF2, and CUC2 [5]. Plant NAC proteins have important functions in modulating the responses to biotic and abiotic stress; for example, more than fifty *NAC* genes participate in plant responses to drought, salinity, and osmotic stresses [6]. Moreover, drought stress induced expression of Arabidopsis *ANAC019*, *ANAC055*, and *ANAC072* and overexpression of these *NAC* genes increased drought tolerance [7]. The rice *NAC* transcription factor gene *OsNAC52* conferred drought tolerance when expressed in transgenic Arabidopsis and activated the expression of abscisic acid (ABA)-related genes [8]. The overexpression of *OsNAC10* enhanced drought tolerance and improved grain yields in rice [9]. *AtNAC2* participates in the responses to various plant hormones, salt stress responses, and lateral root development [10]. To date, only three *NAC* genes have been revealed to respond to high temperature stress in plants. For example, overexpression of rice *ONAC063* in Arabidopsis leads to significant thermotolerance, although high temperature does not increase *ONAC063* expression [11]. Overexpression of Arabidopsis *ANAC042* also improved tolerance to heat stress in transgenic plants [12]. *CsNAM* also responds to heat stress in tea (*Camellia sinensis*) [13]. Therefore, NAC transcription factors may have potential applications in improving biotic and abiotic stress tolerance in plants; however, knowledge of their function in heat stress responses remains limited.

The wheat genome contains multiple *NAC* genes with diverse expression patterns and roles in the responses to environmental factors. Two *NAC* genes (*GRAB1* and *GRAB2*) act in resistance to wheat dwarf virus [14] and *TaNAM-B1* controls the distribution of nutrients between leaves and developing grains to modulate grain protein, zinc, and iron contents [15]. The expression of *TaNAC2* significantly increased in response to salt, low temperature, and drought stresses, and its overexpression in Arabidopsis enhanced tolerance to drought, salt, and freezing stresses by upregulating abiotic stress-responsive genes [16]. *TaNAC69* acts in regulating genes upregulated by stress, and also acts in adaptation to drought stress [17]. *TaNAC67* responds to water deficit, high salinity, low temperature, and ABA treatments, and its overexpression in Arabidopsis can significantly enhance tolerance to drought, salt, and freezing stresses [18]. However, knowledge regarding the functions of wheat *NAC* genes in heat stress responses remains scarce.

Our previous analysis identified 93 wheat *NAC* transcription factor genes that were differentially expressed in response to heat stress (unpublished data). Among these genes, heat stress significantly upregulated *TaNAC2L*, which has high nucleotide sequence similarity to *TaNAC2*. In this study, we examined the roles of *TaNAC2L* in heat stress tolerance. We observed that high temperature increased *TaNAC2L* expression and overexpression of *TaNAC2L* in Arabidopsis enhanced heat tolerance, activating expression of heat-related genes. Post-translational mechanisms also affected the abundance of TaNAC2L. Thus, TaNAC2L affects gene expression in heat tolerance and might be degraded via a proteasome-mediated pathway.

## Materials and Methods

### Plant materials

Two wheat (*Triticum aestivum* L.) cultivars, TAM107 (heat tolerant) and Chinese Spring (CS) (heat susceptible), were planted in a greenhouse at a relative humidity of 75% and a 26/20°C day/night temperature cycle with a light intensity of 3000 lx. Three-leaf seedlings were treated at 40°C for 0.5, 1, and 2 h, and then recovered at 22°C for 24 h.

Seeds of *Arabidopsis thaliana* were surface-sterilized in 0.745% sodium hypochlorite and 0.004% Triton X-100 for 15 min and then washed six times with sterilized water. Sterilized seeds were maintained in darkness for 3 days at 4°C and were then plated on Murashige and Skoog medium. After 7–10 days, plants were potted in soil and grown in a controlled environmental chamber at 22°C and 70% relative humidity with a 16-h light/8-h dark photoperiod.

### Quantitative real-time PCR

Total RNA extraction and first-strand cDNA synthesis was conducted as previously described [19]. Quantitative real-time PCR (qRT-PCR) was performed as follows: 94°C for 3 min; followed by 40 cycles of 94°C for 15 s, 60°C for 20 s, 72°C for 20 s; and then 72°C for 5 min. The quantification results were determined using the 2^_ΔCT^ method according to the formula: ΔCT = CT_Target_−CT_Actin_. For each sample, PCR was performed in triplicate and the mean values of 2^_ΔCT^ were used to determine the differences in gene expression.

### Hypocotyl length measurements

To assay hypocotyl length, the surface-sterilized Arabidopsis seeds were sown on plates containing Murashige and Skoog (MS) medium, maintained at 4°C for 3 days and then incubated upright in a growth chamber at 22°C for 2.5 days in darkness. Seedlings were: 1) maintained at 22°C; 2) treated at 38°C for 90 min; 3) treated at 45°C for 2 h; 4) first treated at 38°C for 90 min, then recovered at 22°C for 2 h followed by 2 h at 45°C [20]. Seedlings were returned to 22°C for 2.5 days and then photographed.

### Arabidopsis transformation

The coding region of the *TaNAC2L* cDNA was amplified from wheat cDNA and cloned into the pEarlyGate201 vector as an HA-fused fragment driven by the CaMV 35S promoter. Transformation vectors harboring the *35S*::*TaNAC2L-HA* construct were introduced into *Agrobacterium tumefaciens* strain GV3101 and then transferred into wild type Arabidopsis plants using the floral dip transformation method. Positive transgenic lines were first screened on Basta plates and then identified by reverse-transcription PCR.

### Western blot analysis

Seedlings were grown in Murashige and Skoog (MS) medium, and seven-day-old seedlings were treated at 38°C for 2 h, then transferred to dimethylsulfoxide (DMSO) for 0.5, 1, 2, 3, 4, 6, or 8 h or to DMSO with 50 μM MG132 for 1, 3, 4, 6, 8, 12, or 24 h before sampling. Plant samples were extracted with extraction buffer (4X SDS loading buffer containing 20 mM Tris-HCl pH 6.8, 40% glycerol, 8% SDS, 20% β-mercaptoethanol (BME) and a small amount of bromophenol blue). The extracts were boiled for 10 min and then centrifuged at 12,000 x *g* for 10 min; the proteins were isolated in the supernatant.

Proteins (10 μl) were separated by SDS-PAGE (10%) and transferred to polyvinylidene difluoride membrane (Millipore), which was blocked with 5% fat-free milk and 0.1% Tween 20 in TBST buffered saline for 1 h, incubated with a 1:3000-diluted antibody against HA (#T506, SAB), washed three times for 5 min each with TBST buffer containing 0.1% Tween 20, and incubated for 1 h with a 1:5000-diluted secondary antibody. The membrane was washed five times, each for 8 min, with TBST buffer containing 0.1% Tween 20 solution before the next step. Specific protein bands were visualized by using Immobilon Western Chemiluminescent horseradish peroxidase substrate.

### Primers

All of the primers used in this study are listed in S1 Table.

## Results

### Wheat *TaNAC2L* transcript levels increased in response to heat stress

**Fig 1:**
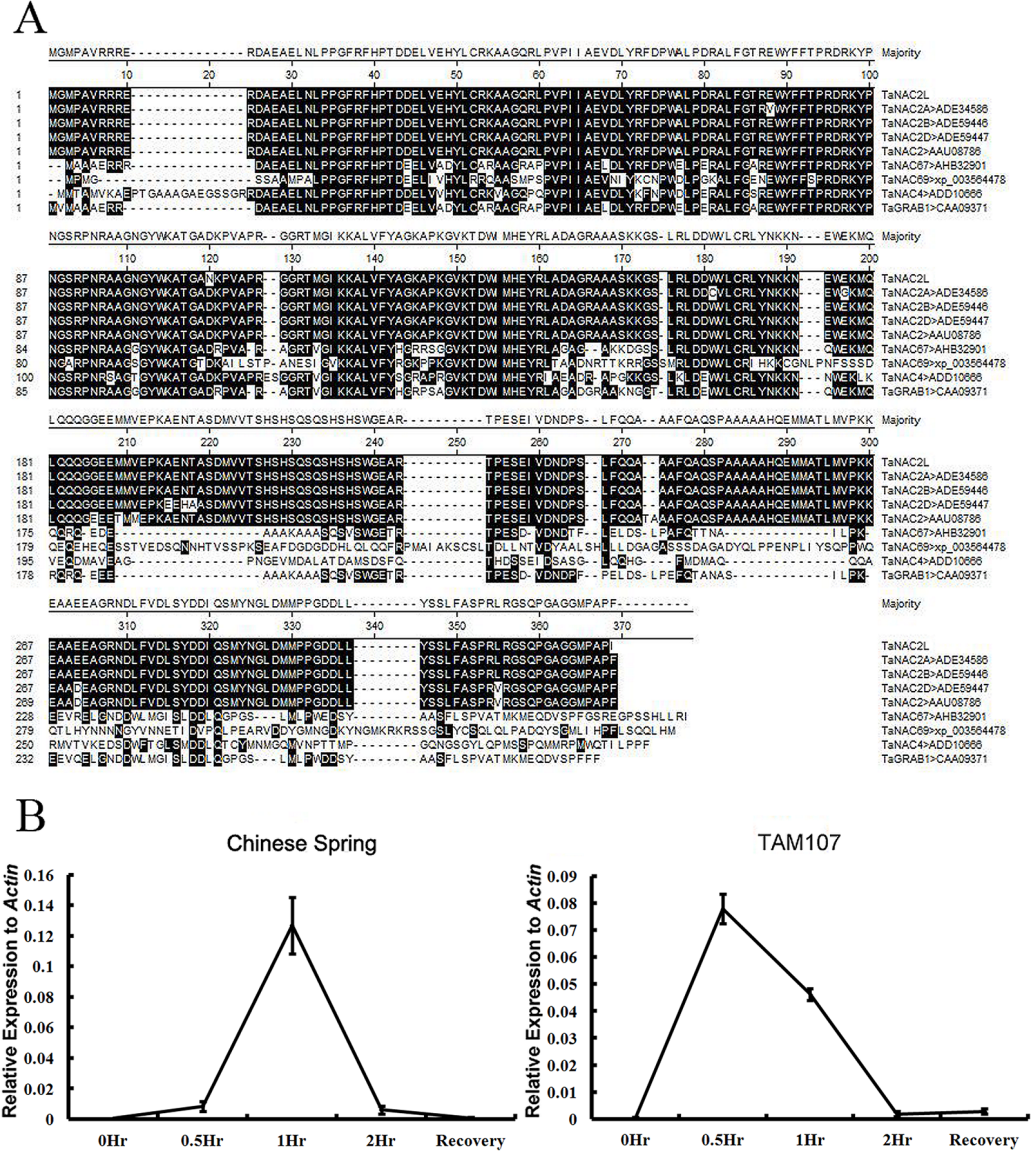
Sequence alignment of TaNAC2L to wheat NAC proteins and *TaNAC2L* expression in response to heat stress. A. Amino acid alignment of TaNAC2L and eight other wheat NAC proteins. The black boxes indicate identical residues and the underlined region indicates the conserved NAC domain. B. Expression of *TaNAC2L* under heat stress. Three-leaf seedlings of the common wheat cultivar Chinese Spring (CS) and the heat-tolerant cultivar TAM107 were exposed to a 40°C high temperature treatment and then recovered in normal conditions after 2 h of heat stress.

Our previous high-throughput sequencing analysis identified one transcript that exhibited significant upregulation after heat stress. The amino acid sequence of this transcript was aligned to known wheat NAC proteins and the results indicated that it differed from TaNAC2, TaNAC2A, TaNAC2B, and TaNAC2D by only 3–4 amino acids; we named this transcript *TaNAC2L* (Fig 1A). We next used BLAST to compare *TaNAC2L* to the assembled wheat genome sequences from IWGSC (http://www.wheatgenome.org/) and found that it showed the highest similarity to the contig on chromosome 5B. *TaNAC2* had been mapped on chromosome 5A [16]; thus, these results indicated that *TaNAC2L* is a homoeolog of *TaNAC2*. To further investigate the expression of *TaNAC2L* during heat stress, real-time quantitative PCR was performed after exposing the heat-sensitive wheat cultivar Chinese Spring (CS), and the heat-tolerant cultivar, TAM107, to normal growth conditions or a 40°C high-temperature treatment. As shown in Fig 1B, transcripts of *TaNAC2L* were present at extremely low levels in both the CS and TAM107 cultivars under normal growth conditions and the response to high temperature differed between the two cultivars. In the heat-sensitive cultivar CS, *TaNAC2L* transcript levels peaked at 1 h after heat stress and then returned to normal levels after the plants recovered. In the heat-tolerant cultivar TAM107, *TaNAC2L* peaked earlier, at 0.5 h after heat stress, and then decreased gradually until 2 h. We speculate that the rapid increase in *TaNAC2L* transcript abundance might be associated with heat tolerance in TAM107.

### Overexpression of *TaNAC2L* in Arabidopsis improved heat stress tolerance via acquired thermotolerance

**Fig 2:**
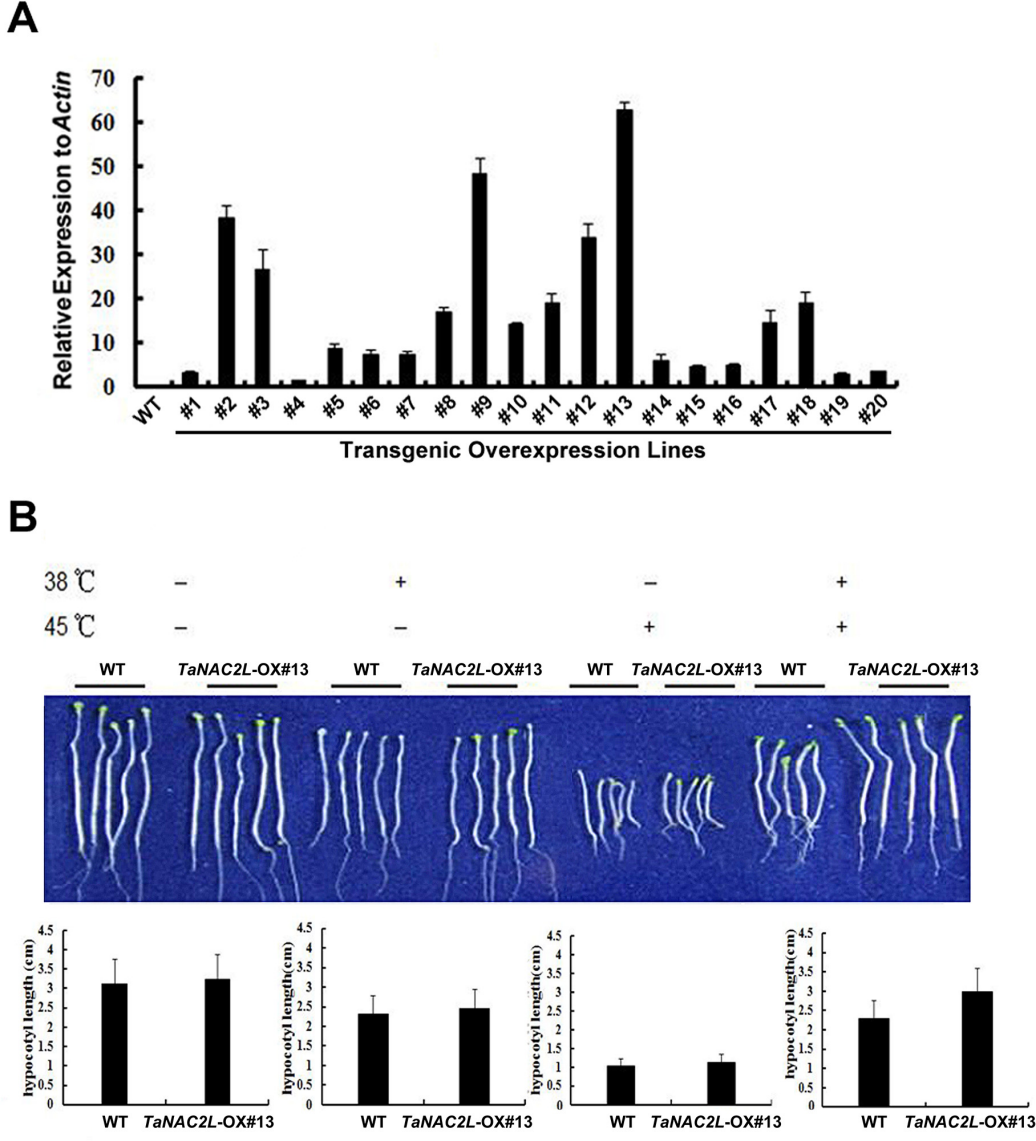
Hypocotyl elongation of *TaNAC2L*-overexpressing transgenic Arabidopsis plants. A. Levels of the *TaNAC2L* transcript in the wild type (WT) and 20 transgenic Arabidopsis lines according to real-time quantitative PCR analyses. B. Hypocotyl elongation of WT and transgenic plants (#13) after growth for 2.5 days in the dark at 22°C. Seedling treatments included: 1) maintained at 22°C; 2) treated at 38°C for 90 min; 3) treated at 45°C for 2 h; 4) first treated at 38°C for 90 min followed by 2 h at 22°C and then 2 h at 45°C. For all treatments, seedlings were returned to 22°C for 2.5 days and then photographed. The quantitative analysis of the hypocotyl length of the wild-type and transgenic plants is presented below the photographs.

To investigate the function of *TaNAC2L* in heat tolerance, we generated transgenic Arabidopsis plants that overexpress *TaNAC2L*. The TaNAC2L produced from this construct was tagged with hemagglutinin (HA) at the N-terminus and expressed under the control of the 35S promoter. A total of 20 transgenic lines were obtained and the expression levels of *TaNAC2L* were determined. Three homozygous transgenic lines (*TaNAC2L*-OX #13, #9, and #2) with higher expression levels of *TaNAC2L* were investigated further (Fig 2A).

Considering that *TaNAC2L* is heat-inducible, we developed a quantitative assay for evaluating the thermotolerance of the transgenic lines compared to the wild type, based on hypocotyl elongation in the dark. We observed no obvious differences between the transgenic lines and wild type Arabidopsis plants after 2.5 days of growth under normal conditions. The three lines with higher expression levels of *TaNAC2L* also showed similar phenotypes after heat stress; thus, only *TaNAC2L*-OX#13 is shown in Fig 2B. The transgenic and wild-type seedlings were exposed to 38°C for 90 min. Wild-type and transgenic plants produced shorter hypocotyls and both were killed by a 2 h treatment at 45°C (Fig 2B). However, when the seedlings were first pre-treated at 38°C for 90 min before exposure to 45°C, the *TaNAC2L*-OX#13 plants produced a longer hypocotyl than the wild-type plants. These results indicate that TaNAC2L promotes a heat stress response via acquired thermotolerance.

### TaNAC2L-regulated stress-responsive gene expression in Arabidopsis

**Fig 3:**
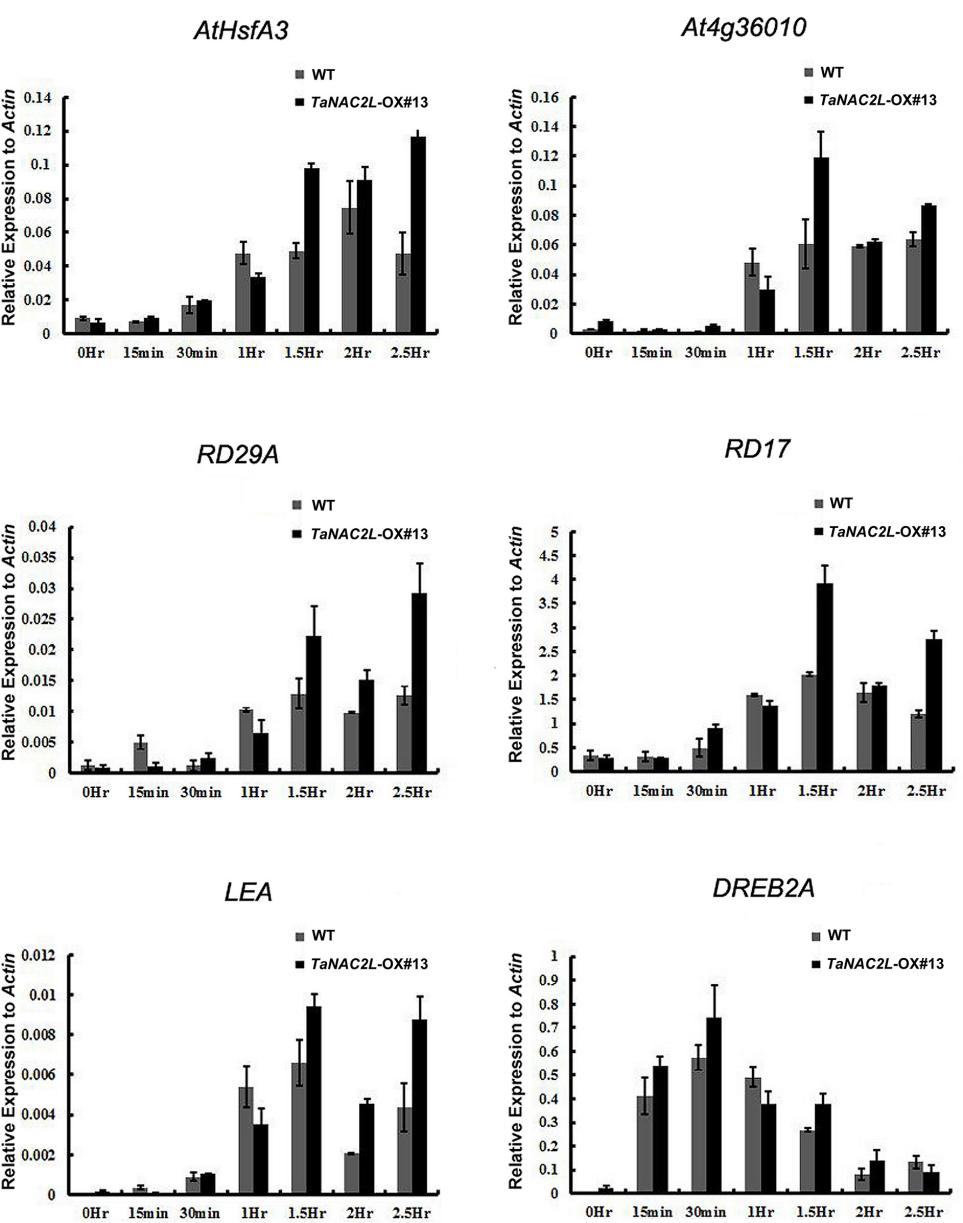
Expression of six stress marker genes in *TaNAC2L* transgenic Arabidopsis plants and wild type (WT) under normal and heat stress conditions. The transcript levels of *DREB2A*, *DREB2B*, *AtCYP18-1*, *RD17*, *HSP26*.*5*, *LEA*, *AtGolS1*, *HSP70*, *AtHsfA3*, and *RD29A* were determined by quantitative RT-PCR. Values represent the mean ± SD of three independent experiments and were normalized to *ACTIN*.

We observed an enhanced tolerance to heat stress in *TaNAC2L*-OX#13 plants compared to wild-type plants. The fact that NAC family proteins may function as transcriptional regulators prompted us to evaluate the changes in the expression levels of stress-responsive genes in the *TaNAC2L*-OX#13 line under heat stress. We measured the expression of 10 stress marker genes: *DREB2A* (At5g05410), *DREB2B* (At3g11020), *AtCYP18-1* (At1g01940), *RD17* (At1g20440), *HSP26*.*5* (At1g52560), *LEA* (At1g52690), *AtGolS1* (At1g56600), *HSP70* (At3g12580), *AtHsfA3* (At5g03720), *RD29A* (At5g52310), and At4g36010 (encoding a thaumatin superfamily protein) in the *TaNAC2L*-OX#13 line and the wild type. As shown in Fig 3, among the ten stress marker genes, six exhibited higher expression levels in the *TaNAC2L*-OX#13 line compared to the wild type from 1.5 h to 2.5 h. These results imply that TaNAC2L may confer heat stress tolerance by regulating stress-responsive genes.

### TaNAC2L was degraded via the 26S Proteasome Pathway

**Fig 4:**
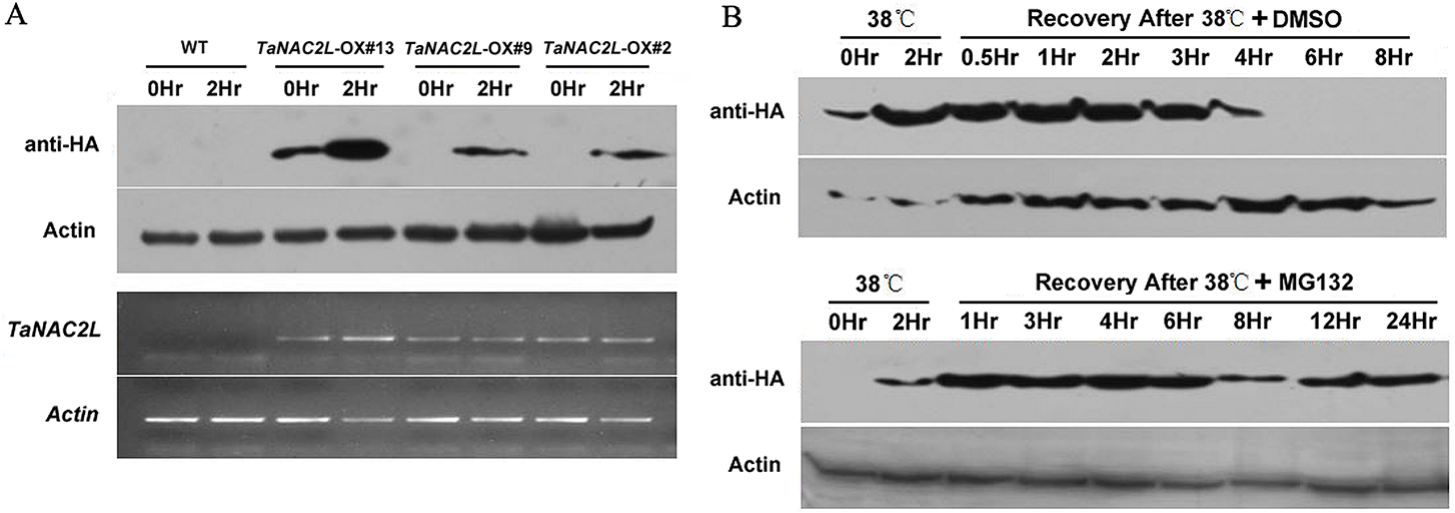
Protein levels of TaNAC2L in *TaNAC2L-OX* transgenic lines. A. TaNAC2L protein and transcript levels in *TaNAC2L*-overexpressing lines (#2, #9 and #13) under normal conditions and 38°C heat stress. Western blot analyses were performed with the anti-HA monoclonal antibody. B. Seven-day-old wild-type (WT) and *TaNAC2L-OX*-#13 seedlings were treated at 38°C for 2 h. After heat treatment, seedlings were transferred to dimethylsulfoxide (DMSO) for 0.5, 1, 2, 3, 4, 6, or 8 h; or DMSO with 50 μM MG132 for 1, 3, 4, 6, 8, 12, or 24 h before harvesting. The HA antibody was used for the immunoblot analyses.

In wheat, heat stress induces *TaNAC2L* expression; however, in the transgenic Arabidopsis lines, *TaNAC2L* was expressed from the constitutive 35S promoter. These transgenic lines exhibited clear transcription of *TaNAC2L*, driven by the constitutive 35S promoter under both normal and heat stress conditions (Fig 4A). To test whether these high, stable *TaNAC2L* transcript levels would affect the abundance of TaNAC2L protein in Arabidopsis, we determined the TaNAC2L protein levels by performing HA immunoblot assays on the three *TaNAC2L*-OX transgenic lines with and without exposure to heat stress. The immunoblot analysis demonstrated that under normal conditions, the TaNAC2L protein was absent in the transgenic lines *TaNAC2L*-OX #2 and #9, and only expressed at a low level in the transgenic line *TaNAC2L*-OX #13. By contrast, after heat stress, the immunoblotting detected a strong protein band in these three independent *TaNAC2L*-OX transgenic lines in the corresponding size range (Fig 4A). Thus, these transgenic lines exhibited clear transcription of *TaNAC2L*, driven by the constitutive 35S promoter, under both normal and heat stress conditions (Fig 4A), but showed high levels of TaNAC2L protein only in response to heat stress. These results suggest that the level of TaNAC2L protein is regulated post-translationally. We further evaluated the TaNAC2L protein level during recovery from 2 h of heat stress. The results indicate that TaNAC2L levels increased in response to the 38°C treatment and decreased gradually with recovery from heat stress (Fig 4B). To further test if TaNAC2L degradation in Arabidopsis was ubiquitination-dependent, we treated the *TaNAC2L*-OX transgenic lines with the 26S proteasome inhibitor MG132. MG132 treatment blocked TaNAC2L protein degradation during recovery from heat stress, suggesting that TaNAC2L might be degraded via a proteasome-mediated pathway.

### TaNAC2L degradation was independent of DRIP1

**Fig 5:**
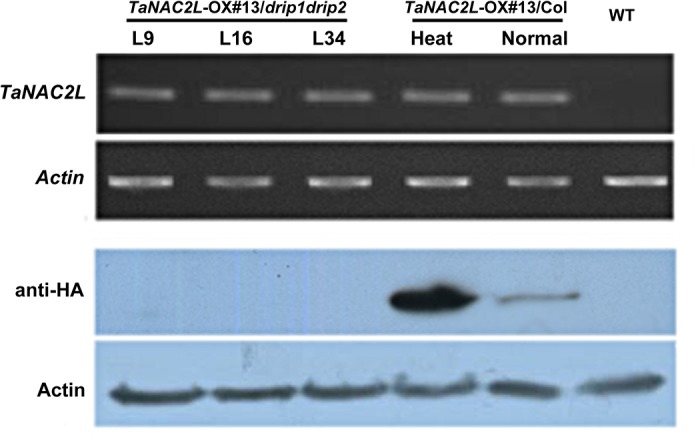
Transcript and protein levels of TaNAC2L in the *drip1 drip2* double mutant background. *TaNAC2L* was highly transcribed in three independent siblings of *TaNAC2L*-overexpressed lines crossed with the *drip1 drip2* double mutant; the absence of DRIP1 and DRIP2 failed to block the degradation of the TaNAC2L protein.

The C3HC4 RING–domain containing proteins DREB2A-INTERACTING PROTEIN1 (DRIP1) and DRIP2 control DREB2A abundance by regulating DREB2A degradation via their function as E3 ubiquitin ligases [21]. This finding prompted us to evaluate whether DRIP1 and DRIP2 regulate TaNAC2L protein degradation. We crossed the *TaNAC2L*-OX#13 transgenic line with a *drip1 drip2* double mutant and measured the levels of TaNAC2L protein. *TaNAC2L* was highly transcribed in the *drip1 drip2* double mutant, but degradation of the TaNAC2L protein occurred normally in the absence of DRIP1 and DRIP2 (Fig 5). These results suggest that other E3 ubiquitin ligases mediate the degradation of TaNAC2L.

## Discussion

Plant NAC transcription factors act in many developmental processes, including in the shoot apical meristem [22] and lateral roots [10, 23], as well as in pattern formation in flowers [24] and in the embryo [25]. NAC transcription factors also affect secondary wall development [26] and leaf senescence [27]. NAC proteins play roles in regulating the transcriptional responses to drought, salt, and osmotic stresses. However, our knowledge of NAC function in the high temperature response of wheat is limited. In this study, we evaluated the potential for using TaNAC2L to improve the tolerance of wheat to heat stress.

*TaNAC2*, *TaNAC2A*, *TaNAC2B*, and *TaNAC2D* participate in the wheat responses to drought, salt, cold, and ABA treatments. Transgenic Arabidopsis lines overexpressing *TaNAC2–GFP* controlled by the 35S promoter exhibited improved tolerance to drought, salt, and freezing stresses and enhanced expression of abiotic stress-response genes [16]. Therefore, TaNAC2 may have potential uses in breeding or transgenic approaches to mitigate the effects of abiotic stress on crops, including dicots. However, its function and mechanism of action during heat stress responses remain unknown. *TaNAC2L*, a putative homoeolog of *TaNAC2* described in this study, differs from TaNAC2 by only a few amino acid variations. We have provided evidence that *TaNAC2L* is induced in heat-stress-sensitive and heat-stress-tolerant cultivars and that the overexpression of *TaNAC2L* enhances acquired heat tolerance by upregulating downstream stress-responsive genes. Our results broaden the knowledge base of *TaNAC2* function in heat-stress tolerance. *TaNAC2*-transformed Arabidopsis plants have longer primary roots and earlier flowering times compared with the wild type [16]; however, the overexpression of *TaNAC2L* in Arabidopsis did not cause any observable phenotypic effects. Hence, we speculate that *TaNAC2L* is a suitable candidate for the improvement of heat stress tolerance in wheat breeding, and possibly in transgenic approaches in other crops.

NAC transcription factors can be regulated at the post-translational level by mechanisms such as ubiquitin-mediated protein degradation [28] and dimerization [29] as well as interactions with other non-NAC proteins [30]. For example, SnRK1 kinases interacted with E3-like ligases and phosphorylated NAC ATAF1, which was then degraded by ubiquitin-mediated proteasomal degradation [31]. In this study, we observed that the TaNAC2L protein levels increased after exposure to heat stress and decreased during the recovery from heat stress, and that the decrease was blocked by MG132. These results suggest that TaNAC2L might be regulated via a proteasome-mediated pathway. Further research is required to identify the proteins that directly or indirectly regulate NAC2L induction and degradation in response to heat stress and recovery.

## Supporting Information

S1 Table Primers used in this study. (DOCX)
