# Supplementary material for: The Wheat NAC Transcription Factor TaNAC2L Is Regulated at the Transcriptional and Post-Translational Levels and Promotes Heat Stress Tolerance in Transgenic Arabidopsis
Source: PLoS One. 2015 Aug 25;10(8):e0135667. doi: 10.1371/journal.pone.0135667 (PMC4549282; doi:10.1371/journal.pone.0135667)
Supplement: S1 Table — (DOCX) [file pone.0135667.s001.docx]

**S1 Table. Primers used in this study.**

| **Primer** | **Primer Sequence（5’-3’）** | **Description** |
| --- | --- | --- |
| ***TaNAC2L*-L1** | CAGAGACAGAGATCGACAGAAG | RT–PCR Primer of *TaNAC2L* for Heat Stress Analysis |
| ***TaNAC2L*-R1** | GAGCTACATCCGCATTGAGAG |  |
| ***TaNAC2L*-L2** | GATGATGGCCACGCTGATG | Primer for quantifying the relative transcript levels of *TaNAC2L* |
| ***TaNAC2L*-R2** | GAGGAGTAGAGCAGGTCGTC |  |
| ***TaNAC2L*-L3** | ATGGGGATGCCGGCGGTGAGG | Primer for cloning the *TaNAC2L* cDNA sequence |
| ***TaNAC2L*-R3** | TTAGAACGGGGCCGGCATGC |  |
| ***AtHsfA3*-L** | TTCGCTAACGAGGCTTTCC | RT-PCR Primers of Stress Response Genes |
| ***AtHsfA3*-R** | CCTCAGTAGGTGACCCTT |  |
| ***At4g36010*-L** | CTTGTGGCGGAGCTGATTAC |  |
| ***At4g36010*-R** | CCTTCGTTGCACTCTTCACA |  |
| ***RD29A*-L** | TGGATCTGAAGAACGAATCTGATATC |  |
| ***RD29A*-R** | GGTCTTCCCTTCGCCAGAA |  |
| ***RD17*-L** | ACGTCCACGCCGTTGGT |  |
| ***RD17*-R** | CTCCGGATGTTCCACTGGAA |  |
| ***AtLEA*-L** | GCAATCAAGAACAAGGCACA |  |
| ***AtLEA*-R** | TCAGTGCGAAGCCCTAAAGT |  |
| ***AtDREB2A*-L** | CAGTGTTGCCAACGGTTCAT |  |
| ***AtDREB2A*-R** | AAACGGAGGTATTCCGTAGTTGAG |  |
| ***TaActin*-L** | GGAATCCATGAGACCACCTAC | RT-PCR Primer of Wheat *Actin* |
| ***TaActin*-R** | GACCCAGACAACTCGCAAC |  |
| ***AtActin*-L** | TGCAGACCGTATGAGCAAAG | RT-PCR Primer of Arabidopsis *Actin* |
| ***AtActin*-R** | CCGTCATGGAAACGATGTCT |  |
